# Epithélioma cunniculatum de topographie inhabituelle

**DOI:** 10.11604/pamj.2015.22.14.680

**Published:** 2015-09-08

**Authors:** Hanane Rachadi, Mohamed Ait-Ourhroui

**Affiliations:** 1 Service de Dermatologie, Vénérologie, CHU Ibn Sina, Rabat, Maroc

**Keywords:** Epithélioma cunniculatum, carcinome verruqueux, tumeur verruqueuse, epithelioma cuniculatum, warty carcinoma, warty tumor

## Image en medicine

Le carcinome verruqueux est une variété de carcinome épidermoïde totalement différencié. Il se développe habituellement au niveau des régions oro-génitales. Il siège rarement au niveau cutané où il est désigné sous le terme d’épithélioma cunniculatum (E.C). R.M âgée de 41 ans avait depuis 4 mois une lésion bourgeonnante de la cuisse, à surface verruqueuse, arrondie, bien limitée, de 3 cm de diamètre. Une résection complète en monobloc de la tumeur était réalisée. L'aspect à l'histologie était en faveur d'E.C. La patiente n'a pas présenté de récidive 2 ans après la résection de la tumeur. L'E.C ou carcinome verruqueux cutané est une tumeur rare, très bien différenciée, à malignité essentiellement locale. Il forme une lésion exophytique, verruqueuse qui peut rester longtemps superficielle. Hormis la localisation palmo-plantaire l'apparition de cette tumeur au niveau des membres n'est rapportée que très rarement dans la littérature. Cette tumeur est sans potentiel métastatique mais elle peut s’étendre aux tissus voisins. Malgré son potentiel d'agressivité locale, les examens histologiques de l'E.C ne révèlent qu'une hyperkératose intense ortho et parakératosique et une dyskératose. Le front d'invasion en profondeur est constitué de bourgeons épithéliaux arrondis soulignés par un infiltrat inflammatoire lymphoplasmocytaire. Il s'y associe un caractère endophytique avec un faux aspect de respect de la membrane basale. Le traitement est mal codifié. Classiquement l'exérèse chirurgicale large et complète s'associe à un taux de guérison élevée. La chimiothérapie complémentaire peut être utile pour prévenir les récidives.

**Figure 1:**
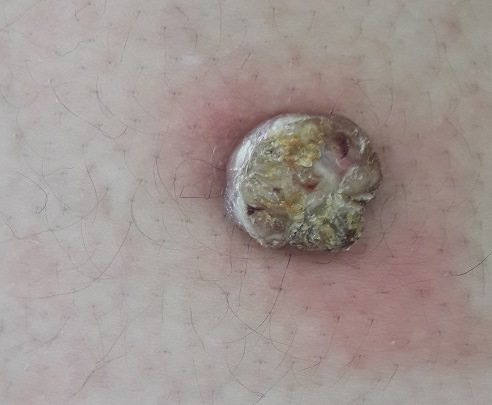
Tumeur verruqueuse de la cuisse

